# ICU readmission: good or bad?

**DOI:** 10.1186/cc9881

**Published:** 2011-03-11

**Authors:** E Potter, D Vondra, S Green, M Zuleika

**Affiliations:** 1 Royal Surrey County Hospital, Guildford, UK

## Introduction

Patients requiring ICU management risk deterioration following discharge. Readmission to the ICU is used as a marker of performance [1] with some controversy [2]. It is established that higher APACHE II scores and longer length of ICU stay are associated with higher risk of ICU readmission [3]. However, there are no criteria available to identify those patients most likely to benefit from readmission [4].

## Methods

Prospective data were collected on all patients admitted to a multidisciplinary adult ICU between 2001 and 2009 and entered into a computerised database. This included length of ICU stay, ICU and hospital outcomes, readmission to ICU and days prior to readmission. Data for all ICU admissions were analysed annually.

## Results

There were 5,004 patients admitted during 2001 to 2009; 1,315 (26%) were elective postoperative admissions and 3,689 (74%) emergency admissions. The ICU mortality during this period was 15.8% and mean APACHE II score was 17.7 (1 to 55). There were 299 readmissions (6%). The average time between discharge and readmission was 8.5 days (0 to 89) with a mean length of ICU stay of 5.89 days (0.2 to 48.8). The average hospital mortality rate of readmitted patients was 33% and fell from 69% in 2003 to 24% in 2007. The proportion of readmitted patients increased from 3% (11) in 2001 to 10% (68) in 2007. As the proportion of patients readmitted increased, the hospital mortality rates for all ICU admissions fell 10% from 31% in 2001 to 21% in 2009.

## Conclusions

As the number of patients readmitted has increased, hospital mortality of both readmitted patients and total ICU patients have fallen. Those readmitted have had a short length of stay (mean 5.89 days).
